# A Prospective Study of Growth and Biomarkers of Exposure to Aflatoxin and Fumonisin during Early Childhood in Tanzania

**DOI:** 10.1289/ehp.1408097

**Published:** 2014-10-17

**Authors:** Candida P. Shirima, Martin E. Kimanya, Michael N. Routledge, Chou Srey, Joyce L. Kinabo, Hans-Ulrich Humpf, Christopher P. Wild, Yu-Kang Tu, Yun Yun Gong

**Affiliations:** 1 Division of Epidemiology, School of Medicine, University of Leeds, Leeds, United Kingdom; 2 Department of Food Science and Technology, Sokoine University of Agriculture, Morogoro, Tanzania; 3 Tanzania Food and Drugs Authority (TFDA), Dar es Salaam, Tanzania; 4 The Nelson Mandela African Institute of Science and Technology (NM-AIST), School of Life Sciences and Bioengineering, Arusha, Tanzania; 5 Institute for Global Food Security, School of Biological Sciences, Queen’s University Belfast, Belfast, United Kingdom; 6 Institute of Food Chemistry, Westfälische Wilhelms-Universität Münster, Münster, Germany; 7 International Agency for Research on Cancer (IARC), Lyon, France; 8 Institute of Epidemiology & Preventive Medicine, College of Public Health, National Taiwan University, Taipei, Taiwan

## Abstract

Background: Aflatoxin and fumonisin are toxic food contaminants. Knowledge about effects of their exposure and coexposure on child growth is inadequate.

Objective: We investigated the association between child growth and aflatoxin and fumonisin exposure in Tanzania.

Methods: A total of 166 children were recruited at 6–14 months of age and studied at recruitment, and at the 6th and 12th month following recruitment. Blood and urine samples were collected and analyzed for plasma aflatoxin–albumin adducts (AF-alb) using ELISA, and urinary fumonisin B_1_ (UFB1) using liquid chromatography–mass spectrometry, respectively. Anthropometric measurements were taken, and growth index *z*-scores were computed.

Results: AF-alb geometric mean concentrations (95% CIs) were 4.7 (3.9, 5.6), 12.9 (9.9, 16.7), and 23.5 (19.9, 27.7) pg/mg albumin at recruitment, 6 months, and 12 months from recruitment, respectively. At these respective sampling times, geometric mean UFB1 concentrations (95% CI) were 313.9 (257.4, 382.9), 167.3 (135.4, 206.7), and 569.5 (464.5, 698.2) pg/mL urine, and the prevalence of stunted children was 44%, 55%, and 56%, respectively. UFB1 concentrations at recruitment were negatively associated with length-for-age *z*-scores (LAZ) at 6 months (*p* = 0.016) and at 12 months from recruitment (*p* = 0.014). The mean UFB1 of the three sampling times (at recruitment and at 6 and 12 months from recruitment) in each child was negatively associated with LAZ (*p* < 0.001) and length velocity (*p* = 0.004) at 12 months from recruitment. The negative association between AF-alb and child growth did not reach statistical significance.

Conclusions: Exposure to fumonisin alone or coexposure with aflatoxins may contribute to child growth impairment.

Citation: Shirima CP, Kimanya ME, Routledge MN, Srey C, Kinabo JL, Humpf HU, Wild CP, Tu YK, Gong YY. 2015. A prospective study of growth and biomarkers of exposure to aflatoxin and fumonisin during early childhood in Tanzania. Environ Health Perspect 123:173–178; http://dx.doi.org/10.1289/ehp.1408097

## Introduction

Poor childhood growth is prevalent in Tanzania, where the proportions of stunting, underweight, and wasting are 42%, 16%, and 5%, respectively, in children < 5 years old [National Bureau of Statistics Tanzania (NBS) and ICF Macro Inc. (ICF Macro) (NBS and ICF Macro 2011)]. Impaired child growth is an indicator of poor nutrition and infection (Black et al. 2013) and is associated with increased susceptibility to disease and mortality, impaired cognitive development, and reduced educational achievement, as well as reduced work capacity and productivity during adulthood [United Republic of Tanzania (URT) and United Nations Children’s Fund (UNICEF) 2010; Victora et al. 2008].

There have been interventions to improve child nutritional status in Tanzania, including vitamin A supplementation and immunization against vaccine preventable diseases, but overall, the prevalence of impaired growth is still unacceptably high (URT and UNICEF 2010). This suggests that other factors in addition to nutrition and infection may have an adverse impact on child growth. To date, any effects that food contaminants such as mycotoxins may have on child growth have not been fully investigated in Tanzania. Mycotoxins are secondary metabolites of fungi that may contaminate crops before or after harvest. Acute and chronic exposure to mycotoxins may cause various human health effects (Sherif et al. 2009). Because these natural contaminants are prevalent in food crops such as the maize and groundnuts that form basic ingredients of complementary foods (i.e., food given to breastfeeding children), children consuming such foods are placed at high risk of exposure to these contaminants. In addition to dietary intake, mycotoxin exposure may also occur *in utero* and through breastfeeding, predisposing children to the risk of chronic exposure from a very early stage of life (Sherif et al. 2009).

Aflatoxins are mycotoxins produced by several species of *Aspergillus* fungi and occur in food crops such as maize, groundnuts, and oil seeds. Aflatoxin B_1_ (AFB1), the most potent aflatoxin, is classified as a human carcinogen [International Agency for Research on Cancer (IARC) 1993], and has been associated with child growth impairment (Gong et al. 2002, 2004; Shouman et al. 2012), suppressed immune function (Turner et al. 2003), hepatomegaly (Gong et al. 2012), and death due to acute poisoning (Probst et al. 2007).

Also of public health concern is another type of mycotoxin that frequently contaminates maize, the fumonisins, produced by several species of *Fusarium* fungi (Shephard et al. 1996). Fumonisin B_1_ (FB1) is the most toxic, and has been classified as “possibly carcinogenic in humans” (IARC 2002). Fumonisin contamination in maize has been associated with esophageal cancer (Rheeder et al. 1992) and neural tube defects (Missmer et al. 2006) in humans, and retarded growth in piglets (Dilkin et al. 2003). Unlike for aflatoxins, for which a number of studies have reported a relationship between exposure and child growth, few epidemiological data are available to date on the relationship between fumonisin exposure and child growth. Only one study has reported an association between fumonisin exposure, measured using a food assessment method, and growth retardation among infants (Kimanya et al. 2010).

Maize is one of the major staple foods in Tanzania and is a basic ingredient in complementary foods for children. Recent studies conducted in some regions of the country have revealed contamination of maize with aflatoxins and fumonisins (Kimanya et al. 2008; Mboya et al. 2011). One of these studies reported that 18% of home-grown maize samples were contaminated with aflatoxins at levels up to 158 μg/kg, with 12% of these above the Tanzanian limit of 10 μg/kg (Kimanya et al. 2008). Fumonisins have been detected in 52% of home-grown maize samples from Tanzania, at concentrations of up to 11 mg/kg, with 15% of these positive samples exceeding 1,000 μg/kg, the maximum tolerable limit for fumonisins in maize for human consumption in other countries (Kimanya et al. 2008). Co-contamination of maize with aflatoxin and fumonisin was reported for 10% of the maize samples tested in that study.

The likely contamination of complementary food ingredients with aflatoxins and fumonisins, and the demonstrated adverse effects on animal and human health, supports the need to investigate whether these mycotoxins are associated with child growth impairment in Tanzania. The objective of the present study was to assess aflatoxin and fumonisin exposures using validated exposure biomarkers, and to estimate their associations with growth.

## Methods

*Subjects and sampling times*. A total of 166 seemingly healthy children (6–14 months old) were randomly recruited from birth registers at local dispensaries and enrolled in this study. See Supplemental Material for “Sample size calculation.”

The children were recruited during maize harvest season when newly harvested maize was consumed, and they were followed-up twice: after 6 months, during the season when stored maize was consumed, and after 12 months, which was another harvest season for maize. During each of the three sampling times, the child’s diet and growth data and samples of blood and urine were collected by trained researchers. The study was conducted in the villages of Nyabula, Kigwa, and Kikelelwa in the respective regions of Iringa, Tabora, and Kilimanjaro in Tanzania. These regions are located in different agro-ecological zones where previous studies showed occurrence of aflatoxins and fumonisins in maize (Kimanya et al. 2008).

*Subjects’ background information*. Children’s birth date, birth weight, sex, and immunization record were obtained from their health clinic cards. For each child, a structured questionnaire was administered to his/her mother or caretaker to collect information on feeding and mother’s basic information. Household socioeconomic status (SES) was calculated using a weighted score based on the type of house building material used for floor, wall, and roof (Bawah and Zuberi 2004).

*Food intake estimation*. During each sampling time, a 24-hr dietary recall questionnaire was administered on two consecutive days to obtain data on details of type and amounts of food given to a child. The questionnaire and procedures followed those previously used for Tanzanian children in communities with similar dietary habits (Kimanya et al. 2009; Mamiro et al. 2005). The mean of the estimated intake for the two recall days was used as the consumption data of each food item. This information was also used to estimate quantities of protein and energy intake per day based on local food composition tables (Lukmanji et al. 2008).

*Anthropometric measurements and growth indices*. Anthropometric measurements of body weight and recumbent length were taken according to World Health Organization (WHO) standardized procedures using calibrated instruments (WHO Multicentre Growth Reference Study Group 2006). Children were weighed in light clothing using a portable spring scale (Salter model 235 6M) and weights were recorded to the nearest 0.1 kg. Recumbent length was measured by SECA 416 infantometer and recorded to the nearest 0.1 cm. Growth indices of length-for-age *z*-score (LAZ), weight-for-age *z*-score (WAZ), and weight-for-length *z*-scores (WLZ) were computed using WHO Anthro software (http://www.who.int/childgrowth/software/en/). Age in months was calculated from difference between date when anthropometric measurements were taken and birth date. Children with LAZ, WAZ, and WLZ scores below –2 SD from the median of the WHO reference population were classified as stunted, underweight, or wasted, respectively (de Onis WHO Multicentre Growth Reference Study Group 2006). Length and weight velocity during the 12 months from the last initial measurements were calculated for each child.

*Biomarkers of aflatoxin and fumonisin exposures*. To provide measures of mycotoxin exposure, blood samples were collected for analysis of aflatoxin exposure biomarker and urine samples for analysis of fumonisin exposure biomarker. During each visit, a single sample of 2 mL venous blood from each child was collected into vacuum collection plain tubes without coagulant (VACUTAINER®) by a qualified nurse at the village dispensary and separated by centrifugation at a local hospital. The first morning urine sample of the child was collected by the mother or child caretaker, using pediatric urine bags (Hollister), after pretraining on how to collect the sample. Two urine samples were collected on 2 consecutive days to obtain a representative estimate of exposure. The urine and blood sample were kept in a cold box on ice immediately following collection, then were kept frozen at –20°C at the Tanzania Food and Drugs Authority (TFDA) before shipping on dry ice to University of Leeds for analysis.

*Analysis of biomarker of aflatoxin exposure*. The biomarker of aflatoxin exposure was plasma aflatoxin–albumin adducts (AF-alb). Levels of AF-alb were determined as described previously (Chapot and Wild 1991). In brief, the procedures involved extraction of albumin, digestion of protein, purification, and ELISA quantification of the AF-alb adducts. Each batch of plasma was analyzed with three positive controls and one negative control for quality control. Measurements were done in quadruplicate on at least two occasions on separate days. The limit of detection (LOD) for the assay was 3 pg/mg of albumin.

*Analysis of biomarker of fumonisin exposure*. Free urinary fumonisin B_1_ (UFB1), a biomarker of fumonisin exposure, was measured as previously described (Gong et al. 2008). In brief, 10 mL of urine was diluted with an equal volume of distilled water, and deuterium-labeled FB1 (FBd6) was added as an internal standard. The FB1 in urine was isolated by solid-phase extraction using Oasis MAX cartridge (Waters). The eluate was dried under vacuum and reconstituted in 200 μL methanol:water (1:1, vol:vol) before injection onto the HPLC-MS (high-performance liquid chromatography–mass spectrometer) (Waters). For quality control, one negative sample and one sample spiked with FB1 were processed together with each batch of urine samples. The mean UFB1 of the 2 days samples was calculated to represent the exposure. The LOD for UFB1 was 20 pg/mL of urine.

*Ethics clearance*. Ethics approval was granted by the National Institute of Medical Research in Tanzania and the University of Leeds (HSLT/09/005). Informed written consent was obtained from the mother of each participating subject.

*Statistical analysis*. Distributions of AF-alb and UFB1 biomarkers were skewed and therefore were natural logarithmic (ln) transformed for statistical analysis. For the data analysis, samples with AF-alb or UFB1 levels below the LOD were assigned half the value of their respective detection limits (Hornung and Reed 1990). Differences in means in mycotoxin exposure biomarkers and growth indices between sampling times and between villages were compared using analysis of variance. The relationship between aflatoxin or fumonisin exposure and child growth was regressed using *a*) biomarker concentrations at recruitment, *b*) the mean concentration of samples collected at recruitment and 6 months later and, *c*) the mean concentration of samples collected at recruitment and at 6 and 12 months after recruitment. Separate multivariable regression models were built with each growth indicator (LAZ, WAZ, WLZ, growth velocity) treated as the outcome variable and aflatoxin or fumonisin biomarker concentrations as predictor covariates. The models were adjusted for village, breastfeeding (partially vs. fully weaned, at baseline survey), maternal education (categorical), and family SES and protein and energy intakes (both as continuous variables). Growth velocity models were additionally adjusted for sex, baseline age in month, and baseline length.

A *p*-value of ≤ 0.05 was considered statistically significant. All statistical analyses were performed using the STATA 11.1 statistical package (StataCorp LP).

## Results

*Demographic characteristics*. Demographic data of the children at the time of recruitment are presented in Table 1. A sample of 166 children (62 in Nyabula, 47 in Kigwa, and 57 in Kikelelwa), 6–14 months of age consisting of 78 boys and 88 girls were enrolled for the study. Follow-up study was not completed for 20 (12%) of recruited children because of outmigration in 12 cases (7%) and withdrawal in 8 cases (5%). Birth weight information was available for only 106 (64%) of the recruited children, of whom 8% had birth weight < 2,500 g. Most children (96%) were from subsistence farming households. About 89% of mothers had completed primary education, and 78% were married. In terms of family SES, Kikelelwa village in the Kilimanjaro region was socioeconomically wealthier than the other villages (*p* = 0.005).

**Table 1:** Characteristics of children and their families at recruitment by village.

Characteristic	Nyabula	Kigwa	Kikelelwa	All villages
Total no. of children	62	47	57	166
Male/female (%)	50/50	36/64	53/47	47/53
Age of child [months (%)]^*a*^
6–9	40	53	67	53
10–14	60	47	33	47
Birth weight [kg (mean ± SD)]^*b*^	2.9 ± 0.5	3.2 ± 0.5	3.4 ± 0.4	3.1 ± 0.5
Age at start of complementary feeding [months (%)]^*c*^
0–3	26	38	60	41
4–5	31	45	31	35
6	43	17	9	24
Partial breastfeeding (%)^*d*^	90	100	91	93
Protein intake/day [g (mean ± SD)]	8 ± 2.0	8 ± 2.4	12 ± 4	9.4 ± 3.5
Energy intake/day [kcal (mean ± SD)]	547 ± 188	615 ± 162	768 ± 236	644 ± 220
Subsistence farming households (%)	98	94	95	96
Mother with primary education (%)	95	77	91	89
Mothers who are married (%)	73	87	75	78
SES score (mean ± SD)	6.0 ± 1.6	6.4 ± 2.3	8.8 ± 1.6	7.1 ± 2.2
^***a***^No significant difference between villages in terms of children’s ages at recruitment. ^***b***^Birth weight data was available for only 64% of subjects. ^***c***^Proportion of children who were introduced into complementary feeding before 6 months old was lower at Nyabula than Kigwa (*p* < 0.05) and Kikelelwa (*p* < 0.001). ^***d***^None of the children were exclusively breastfed.				

*Child feeding*. At recruitment, none of the children were on exclusive breastfeeding. 93% of all the children were on partial breastfeeding (i.e., breastfeeding but also given complementary foods), and this proportion decreased to 78% and 34% after 6 and 12 months from recruitment, respectively. By 6 months of age, 76% of the children were already introduced to complementary feeding (Table 1). Complementary foods were composed mainly of ingredients from locally available food products. Common foods were maize-based porridges, groundnuts, banana, potatoes, rice, finger millet, beans, cassava, meat, fresh cow’s milk, eggs, vegetables, and fruits (see Supplemental Material, Tables S1–S3). The estimated mean protein intake per day at time of recruitment was 9.4 ± 3.5 g, and mean energy intake was 644 ± 220 kcal (Table 1). Mean protein and mean energy intake were significantly higher at 6 and 12 months than at recruitment (*p* < 0.001), but the difference was not statistically significant between sexes.

*Distribution of aflatoxin and fumonisin exposure*. Mean levels of aflatoxin and fumonisin exposure biomarkers by village and sampling time are presented in Table 2. Both AF-alb and UFB1 concentrations are presented as geometric mean with 95% confidence interval (CI) (for further detail, see Supplemental Material, Table S4). At recruitment, AF-alb was detected in 67% of the children (6–14 months old) with a geometric mean concentration of 4.7 pg/mg (95% CI: 3.9, 5.6). The percentage > LOD and geometric mean concentrations increased to 84% and 12.9 pg/mg (95% CI: 9.9, 16.7) after 6 months, and to 99% and 23.5 pg/mg (95% CI: 19.9, 27.7) at 12 months after recruitment, respectively. Both the percentage of positive samples and the mean concentrations were significantly different among the three surveys (*p* < 0.001). At each sampling time, children in Kigwa had higher AF-alb concentrations than children in the other villages.

**Table 2:** Prevalence of detectable^*a *^samples and geometric mean concentrations of AF-alb and UFB1 in children by village and sampling time.

Sampling period	Nyabula	Kigwa	Kikelelwa	All villages	*p*-Value^*b*^
AF-alb
Recruitment
Detectable [*n* (%)]	23 (40)	39 (95)	36 (73)	98 (67)
Geometric mean (95% CI) (pg/mg)	3.0 (2.1, 4.1)	9.3 (7.0, 11.6)	4.6 (3.4, 6.1)	4.7 (3.9, 5.6)	< 0.001
6 months after recruitment
Detectable [*n* (%)]	53 (96)	36 (97)	33 (61)	122 (84)
Geometric mean (95% CI) (pg/mg)	19.9 (13.5, 29.2)	43.2 (28.7, 65.0)	3.6 (2.8, 4.7)	12.9 (9.9, 16.7)	< 0.001
12 months after recruitment
Detectable [*n* (%)]	53 (98)	36 (100)	53 (100)	142 (99)
Geometric mean (95% CI) (pg/mg)	20.8 (16.2, 26.1)	48.8 (34.5, 69.1)	16.1 (12.6, 20.7)	23.5 (19.9, 27.7)	< 0.001
*p*-Value^*c*^	< 0.001	0.011	< 0.001	< 0.001
UFB1
At recruitment
Detectable [*n* (%)]	61 (100)	45 (100)	51 (94)	157 (98)
Geometric mean (95% CI) (pg/mL)	312.2 (230.0, 424.1)	544.2 (397.2, 745.6)	199.7 (137.7, 289.6)	313.9 (257.4, 382.9)	< 0.001
6 months after recruitment
Detectable [*n* (%)]	56 (100)	37 (100)	48 (89)	141 (96)
Geometric mean (95% CI) (pg/mL)	211.7 (161.1, 278.1)	327.2 (217.1, 493.0)	82.8 (58.3, 117.7)	167.3 (135.4, 206.7)	< 0.001
12 months after recruitment
Detectable [*n* (%)]	56 (100)	37 (100)	53 (100)	146 (100)
Geometric mean (95% CI) (pg/mL)	868.3 (617.9, 1220.0)	686.1 (505.4, 931.5)	320.2 (228.9, 448.1)	569.5 (464.5, 698.2)	< 0.001
*p*-Value^*c*^	< 0.001	0.013	< 0.001	< 0.001
^***a***^Detectable refers to AF-alb > 3 pg/mg and UFB1 > 20 pg/mL. ^***b***^*p*-Value for comparing concentrations among villages. ^***c***^*p*-Value for comparing concentrations among sampling times in the specified village.					

At recruitment, UFB1 was detected in 98% of all children at a geometric mean concentration of 313.9 pg/mL (95% CI: 257.4, 382.9). The proportion > LOD and geometric mean concentrations of UFB1 at 6 and at 12 months from recruitment were 96% and 167.3 pg/mL (95% CI: 135.4, 206.7) and 100% and 569.5 pg/mL (95% CI: 464.5, 698.2), respectively. These concentrations differed significantly among the three sampling times (*p* < 0.001). Children in Kikelelwa consistently showed lower levels of UFB1.

*Child growth*. Child growth data by sampling time and village are summarized in Table 3. The overall proportions of stunted children (LAZ below –2) were 44% at recruitment, 55% at 6 months from recruitment, and 56% at 12 months after recruitment, suggesting that there was impairment of growth. There were village differences in mean LAZ scores, with children in Nyabula demonstrating the lowest scores throughout the sampling times (*p* < 0.001). The proportion of underweight children (WAZ below –2) was 8% at recruitment and 14% at both 6 and 12 months after recruitment. At 6 months from recruitment, mean WAZ scores were significantly lower in children in Nyabula than in Kikelelwa village *(p* < 0.05). These levels were also lower in Nyabula than in the other villages at 12 months from recruitment (*p* < 0.01). Overall, WAZ scores were significantly higher at recruitment than at 6 (*p* < 0.01) and at 12 months thereafter (*p* < 0.001). Less than 3% of the children were classified as wasted (WLZ below –2). There were no statistically significant differences in growth index scores according to sex (data not shown).

**Table 3:** Percent of children with growth indices *z*-scores below –2 and distribution of mean *z*-scores.

Growth indices by sampling period	Nyabula		Kigwa		Kikelelwa		All villages		*p*‑Value^*a*^
	*z*‑Score < –2 *n* (%)	Mean *z*‑score ± SD	*z*‑Score < –2 *n* (%)	Mean *z*‑score ± SD	*z*‑Score < –2 *n* (%)	Mean *z*‑score ± SD	*z*‑Score < –2 *n* (%)	Mean *z*‑score ± SD	
At recruitment (*n* = 166)
LAZ	34 (55)	–2.4 ± 1.2	10 (21.3)	–1.2 ± 0.9	29 (51)	–1.8 ± 1.2	73 (44)	–1.9 ± 1.3	< 0.001
WAZ	7 (11)	–0.6 ± 1.2	1 (2)	–0.4 ± 0.9	6 (11)	–0.4 ± 1.3	14 (8)	–0.5 ± 1.2	0.455
WLZ	1 (2)	0.9 ± 1.0	2 (4)	0.3 ± 1.1	0 (0)	0.9 ± 1.0	3 (2)	0.8 ± 1.1	< 0.001
6 months from recruitment (*n* = 148)
LAZ	40 (71)	–2.7 ± 1.3	21 (57)	–1.9 ± 1.0	21 (38)	–1.8 ± 1.1	82 (55)	–2.2 ± 1.2	< 0.001
WAZ	12 (21)	–1.2 ± 1.1	4 (11)	–0.8 ± 0.9	5 (9)	–0.6 ± 1.1	21 (14)	–0.9 ± 1.1	0.027
WLZ	1 (2)	0.2 ± 0.9	1 (3)	0.1 ± 1.0	1 (2)	0.3 ± 1.0	3 (2)	0.2 ± 1.0	0.832
12 months from recruitment (*n* = 146)
LAZ	43 (77)	–2.7 ± 1.2	17 (46)	–1.7 ± 0.9	22 (42)	–1.9 ± 1.1	82 (56)	–2.2 ± 1.2	< 0.001
WAZ	17 (30)	–1.3 ± 1.1	1 (3)	–0.7 ± 0.9	3 (6)	–0.8 ± 0.9	21 (14)	–1.0 ± 1.0	0.002
WLZ	1 (2)	0.1 ± 1.0	0 (0)	0.3 ± 0.9	0 (0)	0.3 ± 0.7	1 (0.7)	0.2 ± 0.9	0.318
^***a***^*p*-Value for comparing *z*-scores between villages.									

**Figure 1:**
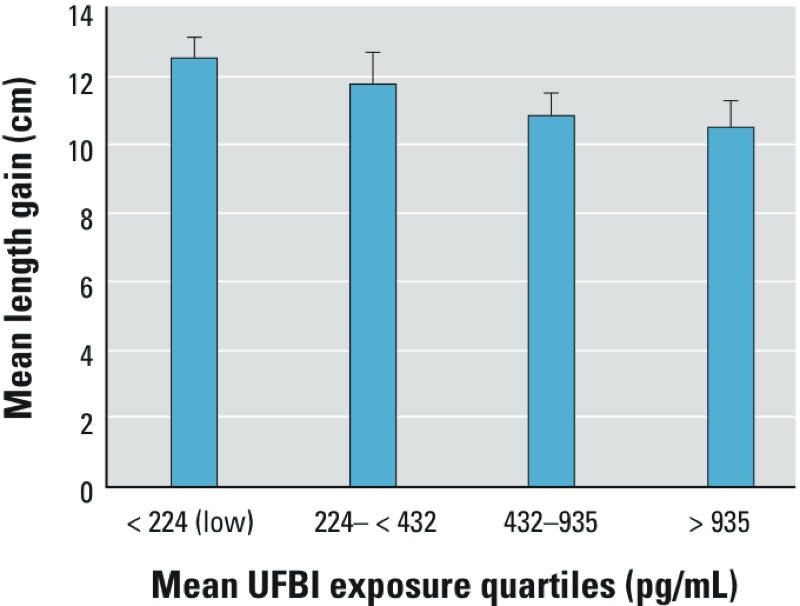
Mean length gain over 12 months, grouped by mean levels of UFB1. Bars show mean length gain (cm) over 12 months, according to exposure quartile groups generated from mean exposure levels from three surveys. Error bars represent 95% CIs. Children in the highest exposure quartile (righthand bar) had significantly less length gained compared with those in the lowest quartile (left-hand bar, *p *= 0.028).

*Association between exposure biomarker levels and growth*. UFB1 concentrations were negatively associated with LAZ but not other *z*-scores at each sampling time (Table 4). Levels of UFB1 at recruitment were negatively associated with LAZ scores at 6 months (β = –0.19; 95% CI: –0.34, –0.04; *p* = 0.016) and 12 months from recruitment (β = –0.20; 95% CI: –0.35, –0.05; *p* = 0.014). The mean UFB1 concentration of the recruitment and 6-month samples was negatively associated with the LAZ scores at 6 months (β = –0.23; 95% CI: –0.41, –0.03; *p* = 0.022) and at 12 months (β = –0.30; 95% CI: –0.45, –0.09; *p* = 0.007) from recruitment, respectively. The mean UFB1 concentration for all three urine samples also was negatively associated with the LAZ scores at 12 months from recruitment (β = –0.39; 95% CI: –0.54, –0.17 for each unit increase in ln-transformed UFB1) In addition, the mean body length gain from recruitment to 12 months was 1.8 cm lower in children with mean UFB1 concentrations (all three samples) in the highest (> 935 pg/mL) versus lowest (< 224 pg/mL) quartile (*p* = 0.028) (Figure 1).

**Table 4:** Multiple regression analyses^*a *^between UFB1 (ln-transformed) and length-for-age *z*-scores (LAZ).

Fumonisin exposure biomarker	*n*	Outcome	Regression coefficient (95% CI)	*p*‑Value
Exposure levels at recruitment	144	LAZ at 6 months from recruitment	–0.19 (–0.34, –0.04)	0.016
	142	LAZ at 12 months from recruitment	–0.20 (–0.35, –0.05)	0.014
Mean exposure levels at recruitment and 6 months after recruitment	147	LAZ at 6 months from recruitment	–0.23 (–0.41, –0.03)	0.022
	145	LAZ at 12 months from recruitment	–0.30 (–0.45, –0.09)	0.007
Mean exposure levels from all three sampling times	146	LAZ at 12 months from recruitment	–0.39 (–0.54, –0.17)	0.000
	146	LAZ scores gained over 12 months	–0.15 (–0.27, –0.02)	0.023
	146	Length velocity over 12 months	–0.52 (–0.91, –0.22)	0.004
^***a***^All models were adjusted for village, breastfeeding, maternal education, SES, and protein and energy intakes. Additionally, length velocity model was adjusted for sex, baseline age, and baseline length.				

Overall, there were nonsignificant negative associations between mean AF-alb levels from all sampling times and LAZ scores at 12 months after recruitment (β = –0.07; 95% CI: –0.27, 0.13; *p* = 0.257), as well as with length velocity (β = –0.33; 95% CI: –0.70, 0.05; *p* = 0.084).

## Discussion

*Child growth*. This study has shown a higher proportion and widespread distribution of stunted growth in these children (i.e., children with LAZ below –2 SD from the mean for the WHO standard reference population) than underweight and wasting. The overall prevalence of stunted growth and underweight observed in the present study is supported by the observations reported for children < 5 years old in the Tanzania Demographic and Health Survey (TDHS) Report of 2010 (NBS and ICF Macro 2011). Mean LAZ and WAZ scores declined with increased age, coinciding with a reduction in breastfeeding as complementary feeding was increased. LAZ and WAZ scores differed among the villages, with the lowest mean scores in children from Nyabula village in the Iringa region. The geographical variation in growth could reflect differences in factors such as SES and child feeding practices. According to TDHS report, Iringa region was one of the four regions in Tanzania with the highest levels of stunting among children < 5 years of age, at 52% (NBS and ICF Macro 2011).

Overall, findings on high prevalence of growth impairment observed in this study highlight the need for local-based strategies for improving children’s nutritional status during the complementary feeding period.

*Distribution of aflatoxin and fumonisin exposure*. Besides the stunted growth that was recorded in about half of the children, the study further revealed high prevalence of aflatoxin and fumonisin exposure. The overall levels of AF-alb across the three sampling times were lower than the geometric mean 32.8 pg/mg AF-alb reported in children 9 months to 5 years of age in Togo and Benin (Gong et al. 2002) and the 31.1–119.3 pg/mg AF-alb in 16- to 37-month-old children from three villages in Benin (Gong et al. 2004). In the present study, the geometric mean plasma AF-alb concentration at recruitment when children were 6–14 months old (4.7 pg/mg) was lower than the geometric mean of 8.7 pg/mg reported for 16-week-old infants in Gambia (Turner et al. 2007). UFB1 concentrations detected at all three sampling times in the present study were lower than the mean of about 3 ng/mL urine recently reported for Cameroonian children (Ediage et al. 2013). However, a limited number of studies have used the UFB1 exposure biomarker in children for comparison. Differences among studies could be attributable to various factors such as differences in age, food contamination levels, food processing and preparation methods, duration of exposure, or individual variation in the toxicokinetics of mycotoxins.

There was disparity in levels of mycotoxin exposure between sampling times, a pattern reflecting the combining effect of increased consumption of contaminated family food, and the seasonal variation of mycotoxins contamination which was previously reported (Turner et al. 2003; Wild et al. 2000). In the present study, AF-alb levels increased progressively, with each sampling time demonstrating higher levels than the previous one. Aflatoxin contamination occurs at both harvest and storage stage due to the nature of the fungus growth and spreading. The observed increase would be expected when stored maize is being consumed because concentrations of aflatoxin increase during storage (Hell et al. 2003). Fumonisin contamination, on the other hand, has been recognized as field-stage only with little increase during storage. Levels of UFB1 were significantly higher at recruitment (maize harvest season) than at the second sampling time, when stored maize was consumed. Reduced fumonisin exposure during the second visit could be explained by reduced maize stocks in the subsistence farming families at 6 months from harvest, which is associated with reduced maize intake and increased reliance on other types of seasonally available local foods. Again, this exposure level became the highest at the last sampling time. Increase in exposure levels with the follow-up time implies that as the children grew older, they were exposed to higher levels due to increased intake of complementary food (Gong et al. 2003).

*Exposure and growth*. In this study, mean AF-alb concentrations from all three sampling times did show a negative association with growth, a direction of effect that is consistent with findings reported in earlier studies in Togo, Benin (Gong et al. 2002, 2004), and Egypt (Shouman et al. 2012). The association, however, did not reach statistical significance in this study. The overall mean of AF-alb across the three sampling times in this study was lower than those reported in children of similar age in the studies cited above, which may explain the nonsignificant nature of association observed. Therefore, this observation from our study does not simply negate the previously established association of aflatoxin exposure with poor child growth. Our findings further demonstrate the rationale for studies to investigate the complicated relationship. It is likely that effects of exposure on growth could also be determined by a number of factors (individually or combined), such as age or specific critical period at exposure (prenatal and/or postnatal), exposure dose, exposure duration, genetics, health, or nutritional status.

Fumonisin exposure appeared to be a possible factor in slowed child growth as levels of UFB1 concentration were negatively associated with growth. The negative association with child growth was consistent when fumonisin exposure was measured either at recruitment or as the mean of two or three time points. This is the first study to report a negative association between fumonisin exposure from biomarker assessment and child growth. A previous study in Tanzania (Kimanya et al. 2010) reported that infants exposed to fumonisins above the WHO provisional maximum tolerable daily intake of 2 μg/kg body weight/day were significantly shorter and lighter than those who were exposed to lower levels (Kimanya et al. 2010). Findings from our study also align with evidence regarding potential mechanisms of effect for fumonisin based on experimental studies, which suggest that this toxin could contribute to growth impairment. Fumonisin disrupts sphingolipid metabolism in the gastrointestinal tract of mice (Enongene et al. 2000), damages intestine permeability in experimental studies (Lallès et al. 2009), and has been associated with decreased food consumption and body weight in piglets (Dilkin et al. 2003). In India, a foodborne disease outbreak in 1995 characterized by diarrhea and abdominal pain was reported to be associated with consumption of maize and sorghum (Bhat et al. 1997). Food samples collected from patients’ households were all positive for fumonisins and contained higher levels of FB1 than those of nonpatients. FB1 was therefore considered to contribute to the outbreak. These findings have raised concern that fumonisin may induce intestinal enteropathy, a subclinical condition of the small intestine, characterized by reduced absorptive capacity and increased intestinal permeability, therefore mediating stunting (Smith et al. 2012).

Although fumonisin exposure was negatively associated with growth, it is important to recognize that the UFB1 biomarker is rapidly eliminated from the urine and thus reflects only very recent exposure (Shephard et al. 1994). Also, there is as yet no proof that FB1 is a direct cause of any chronic health effect, including stunting. It is likely that there are other factors that could affect growth and confound the association between UFB1 levels and child growth seen in this study. Further research is needed to reveal the possible causal mechanisms that could link growth impairment with fumonisin exposure.

## Conclusions

Infants and young children in the study areas were exposed to both aflatoxin and fumonisin. Fumonisin exposure alone, or in combination with aflatoxin, could be among the contributing factors for impaired growth at early childhood and the observed high prevalence of stunting among children. However, causal mechanisms need to be investigated. Appropriate intervention measures to prevent exposure of children to mycotoxins should be considered as one of the key initiatives for improving childhood growth and health in Tanzania and other areas of the world where fumonisin exposure is likely. Studies to investigate specific epidemiological circumstances under which aflatoxin or fumonisin may result in growth defects are recommended.

## Supplemental Material

(225 KB) PDF
