# Erratum: “A Prospective Study of Growth and Biomarkers of Exposure to Aflatoxin and Fumonisin during Early Childhood in Tanzania”

**DOI:** 10.1289/ehp.123-A32

**Published:** 2015-02-01

After Advance Publication of “A Prospective Study of Growth and Biomarkers of Exposure to Aflatoxin and Fumonisin during Early Childhood in Tanzania” (Environ Health Perspect 123:173–178; http://dx.doi.org/10.1289/ehp.1408097), Shirima et al. discovered errors in the models used for analyses of the joint effects of aflatoxin and fumonisin. After they ran the corrected joint-effects models, it became clear that the data were not sufficient to produce interpretable results. Therefore, the authors removed the joint-effects analyses from their manuscript, including information in the Abstract, “Materials and Methods,” and “Results.” Table 5 was also removed, but the main effects of aflatoxins have been added to the “Results.” In addition, Yu-Kang Tu, a biostatistician, was added as a coauthor.

The authors regret the errors.

