# Supplementary material for: A Prospective Study of Growth and Biomarkers of Exposure to Aflatoxin and Fumonisin during Early Childhood in Tanzania
Source: Environ Health Perspect. 2014 Oct 17;123(2):173–8. doi: 10.1289/ehp.1408097 (PMC4314247; doi:10.1289/ehp.1408097)
Supplement: (225 KB) PDF [file ehp.1408097.s001.508.pdf]

Environmental Health Perspectives doi:10.1289/ehp.1408097

<u>Supplemental Material</u>

A Prospective Study of Growth and Biomarkers of Exposure to Aflatoxin and Fumonisin during Early Childhood in Tanzania

Candida P. Shirima, Martin E. Kimanya, Michael N. Routledge, Chou Srey, Joyce L. Kinabo, Hans-Ulrich Humpf, Christopher P. Wild, and Yun Yun Gong

Sample size calculation

Sample size was calculated according to the formula by Daniel, (1999) cited by Naing *et al.* (2006) as follows: - $n = [Z^2 P(1 - P)]/d^2$ whereby; n = sample size, Z = Z statistic for a level of confidence; (1.96 at confidence level of 95%), P = 0.9 (According to earlier study by Kimanya *et al.,* (2008) which found that 10% of infants consumed maize contaminated with both aflatoxins and fumonisins) and d= 0.05 as precision is 5%. Therefore, n=1.96 x 1.96 x 0.9(1 - 0.9)/0.05 x 0.05=138. Assuming 20% possible drop out rate, sample size was adjusted to n= 138+(20/100*138)=166. It was also expected that a group of 166 children with collection of biomarker samples for analysis of mycotoxins exposure and taking growth measurements for 3 surveys would provide a reasonably large number of samples even after dropouts.

Reference

Naing L, Winn T, Rusli B. 2006. Practical issues in calculating the sample size for prevalence studies. Archives of Orofacial Sciences 1(1): 9-14.

Table S1. Types of foods consumed and estimated average intake (g/kg body weight unless otherwise indicated) by all children at recruitment according to village.

Type of food	Nyabula village	Kigwa village	Kikelelwa village
Rice	4.5	5	2.5
Potatoes	0.6	2.1	9.1
Vegetables	8.7	6.1	4.4
Green banana	0	0	13.8
Beans	3.5	3	4.4
Meat	3.9	5	4.3
Fruits	0.5	1.4	0.9
Wheat	0.7	0.4	1.6
Cassava	0.1	0	0.1
Finger millet	1.2	0.9	0.8
Milk[a]	7.5	5.2	15.9

[a]Unit is in mL/kg body weight.

Table S2. Estimated average maize intake by sampling time and village (g/kg body weight/day, mean ± SD).

Sampling time	Nyabula	Kigwa	Kikelelwa	All villages
At recruitment	10.6 ± 4.3*	7.8 ± 4.1	7.7 ± 4.5	8.8 ± 4.5
6 months after recruitment	12.1 ± 5.8*	8.9 ± 5.8	7.9 ± 3.6	9.8 ± 5.4
12 months after recruitment	13.4 ± 4.5	12.9 ± 4.8	9.9 ± 5.7*	12.0 ± 5.3

*At recruitment, maize intake was significantly higher at Nyabula than other villages ($p < 0.01$); at six months from recruitment this intake was higher in Nyabula than Kikelelwa ($p < 0.001$) and Kigwa ($p < 0.01$) while at 12 months from recruitment, the intake was statistically lower at Kikelelwa than Nyabula ($p < 0.01$) and Kigwa ($p < 0.05$).

Table S3. Percent of children consuming groundnuts during the previous week before each sampling time.

Days per week	Nyabula	Kigwa	Kikelelwa
**At recruitment**			
0	81	68	79
1 to 3	13	2	5
4 to 7	6	30	16
**6 months from recruitment**			
0	87	68	85
1 to 3	4	24	7.5
4 to 7	9	8	7.5
**12 months from recruitment**			
0	77	22	75
1 to 3	11	65	17
4 to 7	12	13	8

Table S4. Percentile distribution of geometric mean levels (95% CI) of AF-alb (pg/mg) and UFB1 (pg/mL) by village and sampling time.

Sampling time and village	AF-alb 2.5th percentile	AF-alb 50th percentile	AF-alb 97.5th percentile	UFB1 2.5th percentile	UFB1 50th percentile	UFB1 97.5th percentile
**At recruitment**						
Nyabula	< LOD	< LOD	8.2 (4.3, 15.4)	72.8 (52.8, 100.5)	202.4 (177.1, 231.3)	842.4 (653.9, 1085.3)
Kigwa	< LOD	5.8 (5.1, 6.5)	16.5 (12.6, 21.7)	115.8 (29.5, 455.2)	253.7 (207.6, 310.1)	1297.7 (944.2, 1783.6)
Kikelelwa	< LOD	3.2 (3.1, 3.3)	9.5 (6.7, 13.5)	< LOD	87.6 (68.7, 111.8)	579.0 (421.0, 796.3)
**6 months from recruitment**						
Nyabula	< LOD	6.4 (5.2, 7.9)	65.4 (47.3, 90.2)	27.1 (1.0, 696)	111.1 (92.1, 133.9)	469.9 (350.4, 630.1)
Kigwa	< LOD	20.4 (16.0, 26.0)	110.5 (72.1, 169.4)	30[a]	150.6 (114.9, 197.3)	811.6 (486.2, 1354.9)
Kikelelwa	< LOD	3.2 (3.1, 3.2)	7.4 (5.3, 10.2)	< LOD	40.9 (32.6, 51.4)	229.2 (165.4, 317.6)
**12 months from recruitment**						
Nyabula	< LOD	13.4 (12.2, 14.6)	35.9 (26.9, 47.9)	79.0 (67.2, 92.8)	343.6 (263.0, 448.9)	2437.0 (1841.2, 3225.6)
Kigwa	13.8[a]	23.4 (19.7, 27.7)	104.7 (66.7, 164.2)	126[a]	357.1 (279.7, 456.0)	1448.1 (1089.6, 1925.1)
Kikelelwa	4.2 (0.5, 34.2)	9.3 (8.4, 10.2)	30.5 (21.5, 43.2)	28.1 (3.7, 12.8)	146.1 (116.9, 182.7)	821.0 (572.7, 1176.8)

[a]One observation only; LOD is limit of detection.
